# Competition between Persistent Na^+^ and Muscarine-Sensitive K^+^ Currents Shapes Perithreshold Resonance and Spike Tuning in CA1 Pyramidal Neurons

**DOI:** 10.3389/fncel.2017.00061

**Published:** 2017-03-08

**Authors:** Jorge Vera, Julio Alcayaga, Magdalena Sanhueza

**Affiliations:** Department of Biology, Cell Physiology Center, University of ChileSantiago, Chile

**Keywords:** resonance, oscillations, intrinsic excitability, persistent sodium current, muscarine-sensitive potassium current, hippocampal neurons

## Abstract

Neurons from many brain regions display intrinsic subthreshold theta-resonance, responding preferentially to theta-frequency oscillatory stimuli. Resonance may contribute to selective communication among neurons and to orchestrate brain rhythms. CA1 pyramidal neurons receive theta activity, generating place fields. In these neurons the expression of perithreshold frequency preference is controversial, particularly in the spiking regime, with evidence favoring either non-resonant (integrator-like) or resonant behavior. Perithreshold dynamics depends on the persistent Na^+^ current *I*_NaP_ developing above −70 mV and the muscarine-sensitive K^+^ current *I*_M_ activating above −60 mV. We conducted current and voltage clamp experiments in slices to investigate perithreshold excitability of CA1 neurons under oscillatory stimulation. Around 20% of neurons displayed perithreshold resonance that is expressed in spiking. The remaining neurons (~80%) acted as low-pass filters lacking frequency preference. Paired voltage clamp measurement of *I*_NaP_ and *I*_M_ showed that perithreshold activation of *I*_M_ is in general low while *I*_NaP_ is high enough to depolarize neurons toward threshold before resonance expression, explaining the most abundant non-resonant perithreshold behavior. Partial blockade of *I*_NaP_ by pharmacological tools or dynamic clamp changed non-resonant to resonant behavior. Furthermore, shifting *I*_M_ activation toward hyperpolarized potentials by dynamic clamp also transformed non-resonant neurons into resonant ones. We propose that the relative levels of *I*_NaP_ and *I*_M_ control perithreshold behavior of CA1 neurons constituting a gating mechanism for theta resonance in the spiking regime. Both currents are regulated by intracellular signaling and neuromodulators which may allow dynamic switching of perithreshold behavior between resonant and non-resonant.

## Introduction

Many cognitive and behavioral processes like memory and navigation depend on hippocampal function and rely on network oscillatory activity at frequencies around 4–10 Hz, also known as the theta range (O'Keefe and Recce, 1993; Buzsáki, 2002, 2005; Lisman, 2005). Principal neurons from hippocampal CA1 region are endowed with intrinsic properties that favor activity at theta frequency, displaying an increased subthreshold voltage response for rhythmic stimulation in the theta range, thus acting as resonators (Hu et al., 2002). This selective voltage response or frequency preference is the result of active and passive mechanisms. Specific slowly-activating membrane currents produce an active attenuation of low-frequency voltage responses, while the passive properties of the cellular membrane filter out high-frequency oscillations (Hutcheon and Yarom, 2000). This generates a band-pass filtering effect that tunes the voltage response of resonant neurons around a specific frequency of inputs (Izhikevich, 2002). This frequency selectivity is revealed by a bell-shaped impedance profile that reaches a peak (*Z*_Max_) at the resonant frequency, *f*_R_. Resonance strength is quantified by the Q-value corresponding to the ratio between *Z*_Max_ and the impedance at 0.5 Hz (Hutcheon et al., 1996).

CA1 pyramidal neurons possess two complementary mechanisms to produce subthreshold theta resonance at hyperpolarized or depolarized potentials (Hu et al., 2002, 2009). Below −70 mV, frequency preference is generated by the hyperpolarization-activated cationic current, *I*_h_, a mixed Na^+^, and K^+^ current that activates at membrane potentials below −70 mV (Biel et al., 2009). This current is expressed in an increasing gradient distal from the soma, providing a strong low-frequency filter at dendrites (Magee, 1998).

The mechanism described for subthreshold theta-resonance above −70 mV relay on two voltage-dependent currents widely present in the mammalian brain: the muscarine-sensitive K^+^ current, *I*_M_ (Shah et al., 2002) that produces the actual filter of low frequency oscillations and therefore is called a resonant current (Izhikevich, 2002), and the persistent Na^+^ current, *I*_NaP_, (Crill, 1996) that increases the amplitude of voltage fluctuations acting as an amplifying current (Gutfreund et al., 1995; D'Angelo et al., 1998, 2001).

*I*_M_ is a slow activating/non inactivating voltage-sensitive K^+^ outward current that activates at membrane potentials above ~ −70 mV (Halliwell and Adams, 1982). It depends on the KCNQ2/3 channel, mostly restricted to the somatic region (Wang et al., 1998; Hu et al., 2007).

In turn, *I*_NaP_ is a relatively fast-activating (~1 ms) voltage-sensitive current that activates at potentials above −70 mV, presents a very slow inactivation and is also located in the soma of pyramidal neurons (French et al., 1990; Colombo et al., 2013). This current amplifies subthreshold voltage oscillations in resonant and non-resonant neurons (Hutcheon and Yarom, 2000; Vera et al., 2014). Importantly, elevated *I*_NaP_ levels due to mutations can trigger epileptogenic activity (Kearney et al., 2001), indicating the relevance of *I*_NaP_ in physiological and pathological conditions (Stafstrom, 2007).

Aside the well-documented individual contribution of *I*_M_ and *I*_NaP_ to neuron excitability at subthreshold voltages, the joint contribution of these currents to shape perithreshold dynamics remains poorly understood. The similar voltage range of activation and the opposed effects on membrane potential of these currents, together with a high degree of modulation by second messengers and neuromodulators (Crill, 1996; Marrion, 1997), place the combination of the two currents as a feasible and sophisticated mechanism to control perithreshold dynamics.

Perithreshold resonance is not always observed (discussed in Hu et al., 2002) and the general use of tetrodotoxin (TTX) to avoid contamination of recordings with spikes (and also eliminating *I*_NaP_) occludes the possibility to evaluate if frequency preference is translated to the firing regime (Hu et al., 2002). In addition, it is often considered that hippocampal pyramidal neurons behave only as integrators under *in vitro* conditions (Prescott et al., 2008). Therefore, it is still unclear whether CA1 neurons can express perithreshold resonance and impact firing.

To understand the mechanism that controls perithreshold excitability, we conducted a comprehensive study of the contribution of *I*_M_ and *I*_NaP_ to oscillatory processing using whole-cell recordings in current clamp, voltage clamp, and dynamic clamp, and computational modeling.

We found that CA1 pyramidal neurons display a differential behavior at perithreshold voltage range due to a heterogeneous contribution of *I*_NaP_ and *I*_M_. Reducing *I*_NaP_ or increasing *I*_M_ switches non-resonant neurons to resonant, which suggest that perithreshold frequency preference depends on the dynamic interaction between *I*_NaP_ and *I*_M_.

## Methods

Animal care and experimental procedures were approved by the Bio-Ethical Committee of the Facultad de Ciencias, Universidad de Chile, according to the ethical rules of the Biosafety Policy Manual of the Fondo Nacional de Desarrollo Científico y Tecnológico (FONDECYT), Chile.

### Slice preparation

Male Sprague Dawley rats, from 18 to 30 days-old, were anesthetized and decapitated. The brain was rapidly removed and transferred to an ice-cold dissection solution containing (in mM): 206 sucrose, 2.8 KCl, 1 MgCl_2_, 2 MgSO_4_, 1 CaCl_2_, 26 NaHCO_3_, 1.125 NaH_2_PO_4_, 10 glucose, and 0.4 ascorbic acid (equilibrated with 95% O_2_ and 5% CO_2_), pH 7.3. Septotemporal slices (400 μm) containing dorsal hippocampus, were obtained with a vibratome (Vibratome Sectioning System 102, Pelco, USA). Slices were placed in a holding chamber with standard artificial cerebro-spinal fluid (ACSF) and were left to recover during at least 1 h at 30°C before using them for recordings.

### Electrophysiological recordings

Whole cell patch-clamp recordings were conducted under visual guidance with an upright microscope (Nikon Eclipse E600FN, Nikon Corp. Instruments Co., Japan) equipped with oblique infrared and DIC optics. Electrodes (2.5–3.0 and 3.5–4.0 MΩ for voltage and current-clamp experiments, respectively) were fabricated from borosilicate glass capillary tubing (0.8–1.10 × 100 mm; Kimble Glass Inc., USA) using a horizontal puller (Flaming/Brown P-97, Sutter Instrument Co., USA). Current-clamp and voltage-clamp recordings were made with an EPC-10 patch-clamp amplifier (Heka, Heidelberg, Germany); signals were filtered at 10 kHz and acquired 25 kHz using the Heka Patchmaster software. In voltage-clamp experiments series resistance was compensated by 60–70%. Only cells with a stable resting membrane potential negative to −60 mV were used for recordings. Spike threshold was measured either during stimulation with a current ramp (8 pA/ms; from −80 mV until firing) or during the application of a pseudo-sinusoidal current of linearly increasing frequency and constant amplitude (ZAP stimulus). Threshold was defined as the membrane potential for which the time derivative exceeded 5 mV/ms.

All experiments were performed in presence of 10 μM CNQX and 100 μM PTX to block AMPA-R and GABA_A_-R mediated currents. In some experiments 100 μM APV was also added.

CA1 pyramidal neurons were identified morphologically by their soma located in the stratum pyramidale that extend to stratum radiatum with a prominent apical dendrite. Once in whole-cell configuration was confirmed a resting membrane potential below −70 mV (−81.5 ± 0.8 mV, *n* = 32) and input resistance at −80 mV near 60 MΩ (55.9 ± 2.3 MΩ, *n* = 32).

### Recording solutions (in mM):

Artificial cerebro-spinal solution (ACSF) contained (in mM): 124 NaCl, 2.8 KCl, 1.25 NaH_2_PO_4_, 26 NaHCO_3_, 10 Glucose, 2 MgCl_2_, 2 CaCl_2_, and 0.4 ascorbic acid (equilibrated with 95% O_2_ and 5% CO_2_), pH 7.3 and 285–295 mOsm.

Low Na^+^ ACSF (in mM): 38 NaCl, 80 NMDG, 80 HCl, 2.8 KCl, 1.25 NaH_2_PO_4_, 26 NaHCO_3_, 10 Glucose, 2 MgCl_2_, 2 CaCl_2_, and 0.4 ascorbic acid (equilibrated with 95% O_2_ and 5% CO_2_), pH 7.3 and 285–295 mOsm.

Internal pipette solution was based in previous works reporting stability of resting membrane potential, action potential threshold, and after-potential depolarization (Xu et al., 2005; Kaczorowski et al., 2011), in mM: 123 K-Gluconate, 10 KCl, 4 Glucose, 1 EGTA, 10 HEPES, 2 Na_2_ATP, 0.2 Na_3_GTP, 10 phosphocreatine, 1 MgCl_2_, 0.1 CaCl_2_, and 0.1% biocytin, pH 7.35, and 285–290 mOsm.

### Liquid junction potential (LJP)

We measured the LJP between pipette solution and both ACSF (~13 mV) and low Na^+^ ACSF (~17.5 mV), according to the procedure described by Neher (1992), and recorded values were corrected offline during analyses.

### ZAP stimulation and analysis

Voltage responses to an intracellularly injected pseudo-sinusoidal current of linearly decreasing or increasing frequency and constant amplitude (10 pA) (ZAP stimulus; frequency interval: 0–15 or 20 Hz, 10 s duration) were recorded in current clamp conditions. In experiments with blockers of voltage-dependent channels the amplitude of ZAP stimuli was adjusted to maintain a peak to peak voltage response comparable to the control condition (5–10 mV) and to favor the evaluation of perithreshold resonance in the absence of spikes. In all experiment the protocol was repeated 8–10 times in every neuron, for each condition. The output equipotential subthreshold waves were averaged to proceed with the impedance analysis.

The impedance frequency profile (*Z(f)*) was obtained from the output (voltage) and input (current) waves Fast Fourier Transforms (FFT) ratio (*Z(f)* = *FFT[V(t)]/FFT[I(t)]*). The impedance is a complex quantity (*Z(f)* = *Z, Real* + *Z, Imaginary*), where the real part (*Z, Real*) is the resistance and the imaginary part (*Z, Imaginary*), the reactance. For each given frequency, the complex impedance can be plotted as a vector with magnitude (|*Z(f)*|) and phase. We only focused on the effects on impedance magnitude obtained with the following expression.

(1) $$\begin{array}{r} |Z\left(f\right)|=\sqrt{{\left(Z,\;Real\right)}^{2}+{\left(Z,\;Imaginary\right)}^{2}} \end{array}$$

Throughout the text the term *impedance* will be used to refer to the magnitude of the impedance vector, unless otherwise stated. Frequencies below 0.5 Hz were not plotted in the impedance profiles graphs, to avoid low frequency distortions. Off-line analyses and graphs were performed with Igor Pro 6.2 software (WaveMetrics Inc., USA).

### Quantification of resonance

Resonance is defined as the band-pass filter property of the impedance profile (Hutcheon and Yarom, 2000). The Q factor or value is a measure of resonance strength and is quantified as the ratio between the maximal impedance (i.e., the impedance at the resonance frequency, *|Z(f*_*res*_*)|*) and the impedance at the lowest frequency (|*Z(0.5)*|) (Hutcheon et al., 1996). Here we used Q = 1 in the subthreshold depolarized voltage response as a criterion to define non-resonant behavior. To quantify resonance from recordings containing action potentials we defined an apparent Q value (Q′-value) using as point of reference the impedance at 1 Hz (**Figures 2**, **6**) and 2 Hz (**Figure 7**) instead of 0.5 Hz, to discard suprathreshold contribution of spike firing to impedance profile.

### Firing probability measurements

Firing probability under ZAP stimulation was computed for each oscillatory period as the number of sweeps in which neurons fired a spike divided by the total number of sweeps (typically 8). The frequency of stimulation associated to each depolarized excursion was the frequency at the peak of each current oscillation (starting near 1.2 up to 20 Hz). For instance, if a neuron fired a spike at a given frequency in 4 out of 8 sweeps, its firing probability at that frequency is 0.5.

### Voltage clamp measurement of *I*_NaP_ and *I*_M_

Despite technical limitations of whole-cell voltage clamp technique on neurons with complex morphology, numerous studies have shown that single-electrode voltage-clamp measurement of whole cell currents is suitable for recording in brain slices, including kinetics and voltage sensitivity of *I*_NaP_ and *I*_M_ (Halliwell and Adams, 1982; French et al., 1990; Hu et al., 2009). However, it is necessary to be aware of the lack of space-clamp control and its effects on regions distal to the soma. Three reasons support an acceptable quality of our measurements. First, both *I*_NaP_ and *I*_M_ are conductances located mainly at the soma where space-clamp control is possible. Second, the current injection needed for depolarizing to perithreshold potential is low (below 800 pA), reducing the error of our measurements. In addition, the reduction of Na^+^ driving force together with a slow ramp protocol allows inactivation of *I*_*NaT*_ and a low amplitude and well-clamped *I*_NaP_ recording. And third, most of our conclusions are taken from a relative comparison of *I*_NaP_ and *I*_M_, with both currents sequentially recorded in the same cell, with the same pipette under the same conditions, thus decreasing possible bias due to different experimental configurations.

Furthermore, our voltage clamp data agree with values recorded in dissociated neurons (French et al., 1990), with voltage dependent behavior of neurons recorded in current clamp (**Figures 2**, **3**) and also our voltage clamp data is able to reproduce voltage dependent behavior of neurons when are used to feed the computational models (**Figure 5**).

### Fitting curves

To characterize voltage dependence of *G*_NaP_ and G_M_ we fitted the following sigmoid curve:

(2) $$\begin{array}{r} G_{i}\left(V\right)=G_{Max}\frac{1}{1+e^{\frac{V-V_{0.5}}{s}}} \end{array}$$

*G*_*Max*_ is the maximal conductance, *V*_0.5_ is the voltage for half activation and *s* is the slope of voltage sensitivity. Despite experimental curves for *G*_M_ did not reach saturation, fitting values allowed a good mathematical characterization of conductance curve at the voltage range of interest.

### Computer simulations

To explore perithreshold behavior of neurons recorded under voltage-clamp we developed a point process conductance-based model following the Hodgkin-Huxley equations (Hodgkin and Huxley, 1952). The model included a passive leak current (*I*_Leak_), a persistent (non-inactivating) Na^+^ current (*I*_NaP_) (French et al., 1990) and the slow muscarine-regulated K^+^ current, *I*_M_ (Adams et al., 1982). The model did not include a firing mechanism (fast sodium and potassium conductances).

The equation describing the evolution of membrane voltage (V) in time is

(3) $$\begin{array}{r} C\frac{dV}{dt}=I_{ZAP\;}-\;I_{Leak\;}-I_{\text{M }}-I_{\text{NaP }} \end{array}$$

where *C* is the membrane capacitance estimated for each cell from the capacitive current elicited with a −5 mV step at −70 mV holding potential in voltage clamp configuration (Golowasch et al., 2009), and *I*_*ZAP*_ is the applied current. Intrinsic ionic currents in Equation 3 follow the next set of equations

(4) $$\begin{array}{r} I_{Leak}=G_{Leak}\left(V-E_{Leak}\right) \end{array}$$

(5) $$\begin{array}{l} I_{\text{NaP}}=G_{\text{NaP}}w\left(V-E_{Na}\right) \end{array}$$

(6) $$\begin{array}{r} I_{\text{M}}=G_{\text{M}}r\left(V-E_{K}\right) \end{array}$$

where *G*_Leak_, *G*_M_ and *G*_NaP_ are the maximal conductances of the corresponding currents and *E*_Leak_, *E*_K_, and *E*_Na_ the reversal potentials of *I*_Leak_, K^+^- and Na^+^-mediated currents, respectively, and *r, w* are the state variables (see below). *E*_Leak_ was set to −70 mV, *E*_K_ (−99 mV) and *E*_Na_ (47.1 mV) were calculated from the ionic conditions in our current clamp recordings. To simulate each recorded neuron, maximal conductance and steady-state values for *I*_NaP_ and *I*_M_ were obtained by fitting a sigmoid curve from experimental data (**Figure 4**) as explained above. This gave us a specific set of parameters for each neuron from −70 to −40 mV, allowing a precise characterization of voltage dependence at perithreshold region.

The dynamics of the state variables *x*_*i*_ = *r* and *w* is ruled by the following equation:

(7) $$\begin{array}{r} \frac{dxi}{dt}=\frac{xi_{\infty }\left(V\right)-xi}{\tau_{xi}\left(V\right)} \end{array}$$

where *xi*_∞_ are the steady-state values of *xi*, and τ_*xi*_ are the corresponding time constants.

The time constant for *I*_NaP_ (τ_NaP_) was set at 1 ms according to Vervaeke et al. (2006) and for *I*_M_ was modeled according to the voltage dependent equation (Adams et al., 1982):

(8) $$\begin{array}{r} \tau_{\text{M}}=\frac{1000}{3.3\left(e^{\left(V+35\right)/40}+e^{-\left(V+35\right)/20}\;\right)} \end{array}$$

and was divided by the temperature-correcting factor 3^(*T*−22)/10^ (Hodgkin and Huxley, 1952) to set simulations at 35°C.

Simulations were performed using Igor Pro 6.2 software on a Mac Book Pro (Apple Inc., USA) computer. An integration time step of 10 μs (100 kHz) was used for all simulations. The stimulation protocol used in all the cases comprised sequential ZAP current injections, each of 10 s duration and 5 pA amplitude, and ranging from 0 to 20 Hz. The ZAP injections were superposed to increasing holding current steps that moved the average potential between ~ −70 and −35 mV, as in the electrophysiological current-clamp experiments. The code for reproducing the computer simulations described in this paper is available upon request to authors.

### Dynamic clamp

For dynamic-clamp experiments, the current-clamp amplifier was driven by an analog signal from a desktop computer running Real-Time Linux Dynamic Clamp (Real-Time Experimental Interface, RTXI Dorval et al., 2001; Bettencourt et al., 2008) using an update frequency of 25 KHz.

Dynamic current used to decrease endogenous *I*_NaP_ or increase *I*_M_, were introduced via dynamic clamp using Equations (5) and (6), where *V* is the online measured membrane potential. The dynamics of the state variables *w* and *r* were modeled according to Equation (7). The voltage dependence of the state variables at equilibrium was given by the equations:

(9) $$\begin{array}{r} w_{\infty }=\frac{1}{1+e^{-\left(V+V_{0.5}\right)/5}} \end{array}$$

(10) $$\begin{array}{r} r_{\infty }=\frac{1}{1+e^{\left(V+35\right)/10}} \end{array}$$

where V is the online recorded membrane potential and *V*_0.5_ is the potential for half activation of *I*_NaP_. Here we set *V*_0.5_ as −52 mV according to our average voltage-clamp measurement (**Figure 4**).

The time constant for *I*_NaP_, τ_NaP_, was set at 1 ms according to Vervaeke et al. (2006) and for *I*_M_ was modeled according to Equation (8).

Here we show dynamic currents as the external current that was injected to neurons, following the standard convention were positive currents are depolarizing and negative currents are hyperpolarizing.

### Statistical analysis

**Figure 1:**
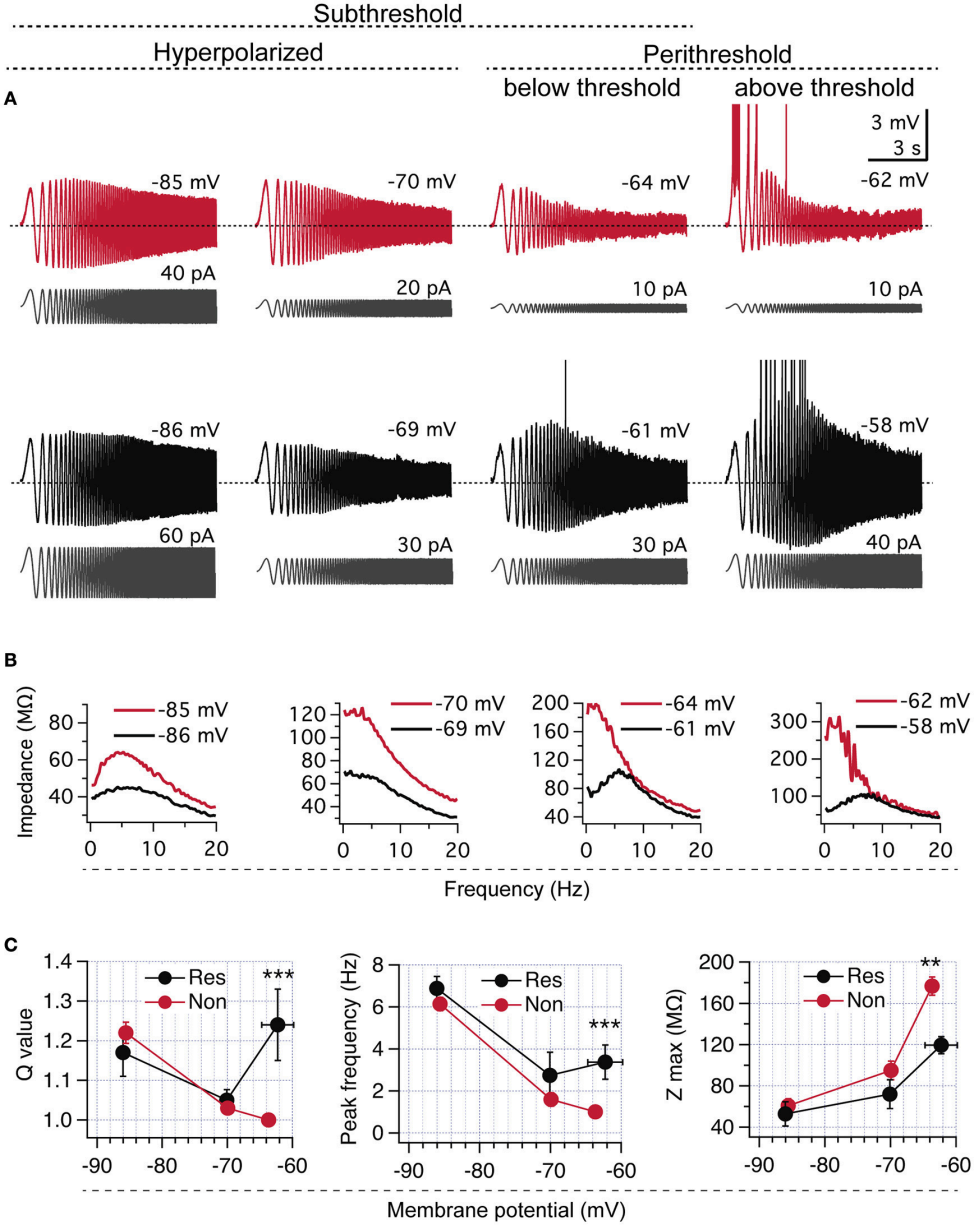
**CA1 pyramidal neurons display two different perithreshold behaviors under oscillatory stimulation. (A)** Voltage responses of two CA1 pyramidal neurons (red and black) stimulated with ZAP protocols (gray) from hyperpolarized (left) to depolarized perithreshold potentials (right; below and above spike threshold). While hyperpolarized responses were similar, cells presented different perithreshold behaviors, non-resonant (red), and resonant (black). **(B)** Impedance profile obtained from recordings in **(A)**. **(C)** Quantification of resonance parameters in the whole subthreshold range for neurons displaying non-resonant (Non, *n* = 21) and resonant (Res, *n* = 5) perithreshold behavior. Mann Whitney test (Q value) and Two-way ANOVA (peak frequency and *Z*_*max*)_. ^**^P < 0.01, ^***^P < 0.001.

**Figure 2:**
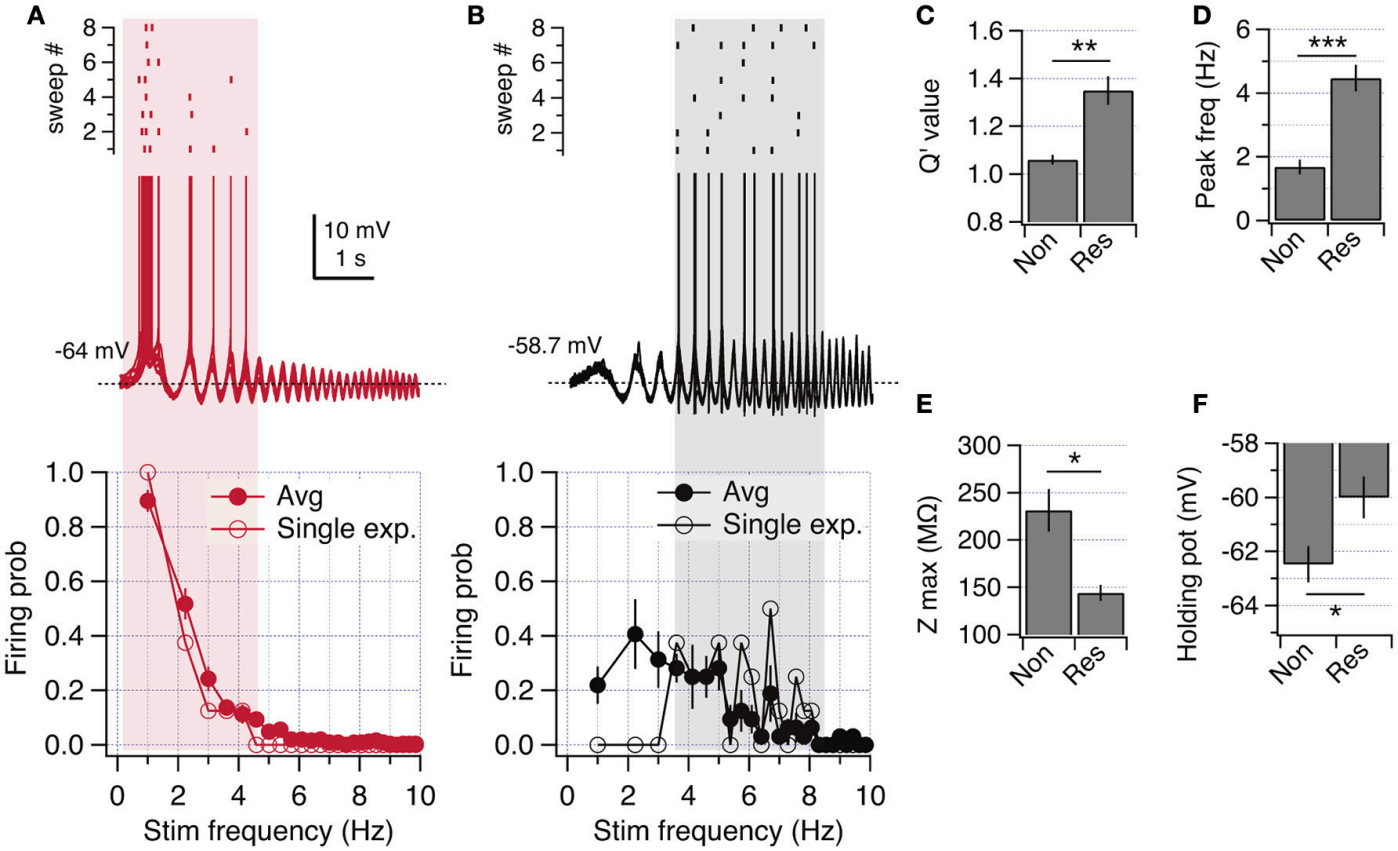
**Resonant and non-resonant behaviors in the spiking regime. (A)** ZAP-induced suprathreshold discharge of a non-resonant neuron (overlay of 8 consecutive recordings). The raster plot shows the firing activity for each trace (top). The firing probability as a function of stimulation frequency is shown at the bottom, including a single curve of a representative neuron, and the average curve (*n* = 21). **(B)** Same as in **(A)** but for a resonant neuron (average curve for *n* = 5). **(C–F)** Quantification of resonance parameters in the spiking regime (see Methods) and other excitability properties of non-resonant and resonant neurons: Q′ value **(C)**, peak frequency **(D)**, peak impedance **(E)** and holding potential at which transition to spiking occurs **(F)**. Mann Whitney test **(C)** or unpaired *t*-test **(D,F)**. ^*^P < 0.05, ^**^P < 0.01, ^***^P < 0.001.

**Figure 3:**
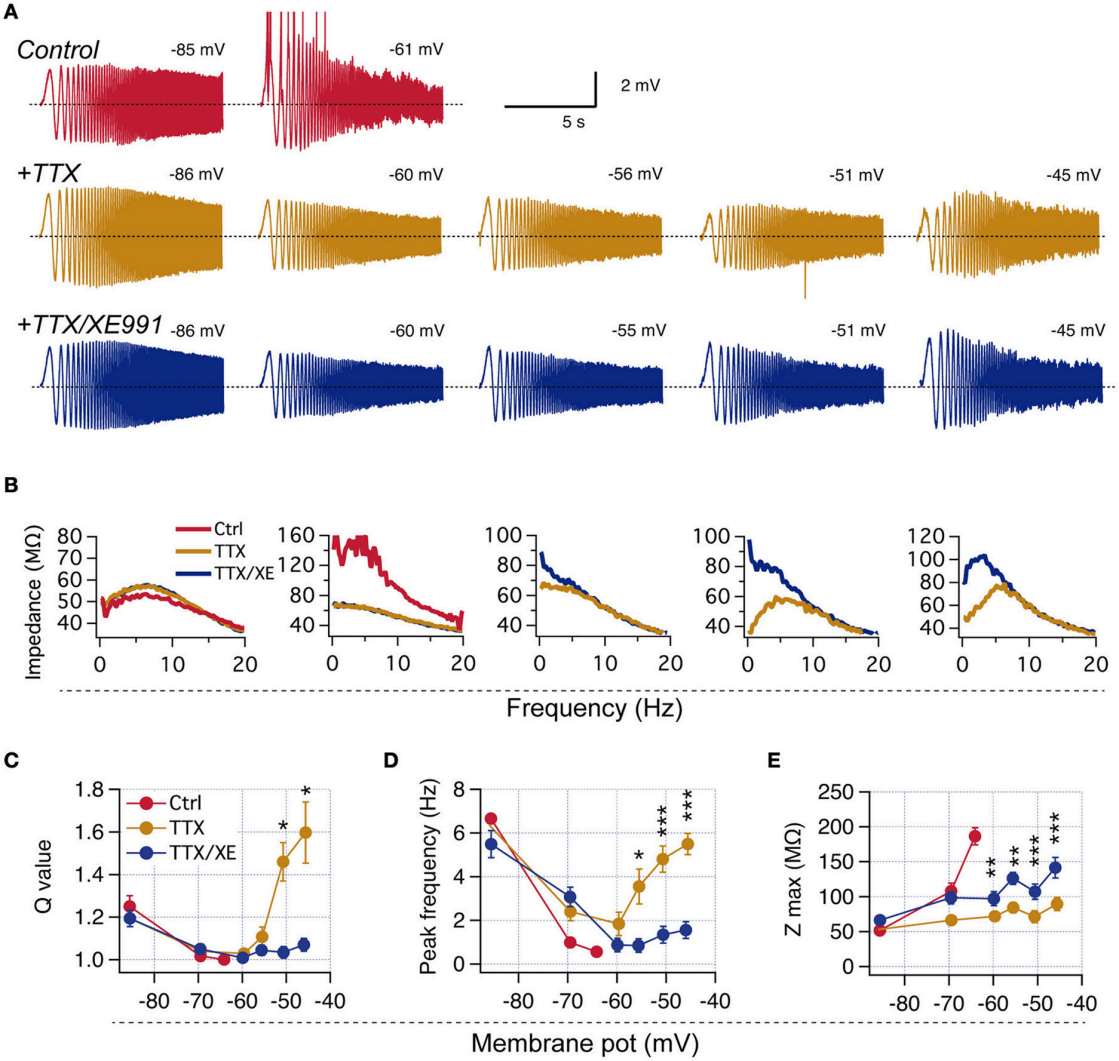
**Neurons lacking perithreshold resonance display M-resonance at more depolarized potentials when spiking is prevented. (A)** Representative experiment showing the expression of M-resonance in a non-resonant neuron when action potentials are blocked with TTX. *Top*, Voltage response to ZAP stimulation at −85 and −61 mV in control condition (red). *Middle*, Recordings from the same neuron at different membrane potentials after addition of 1μM TTX. *Bottom*, Same as before after application of 10 μM XE991. **(B)** Impedance profiles obtained from recordings in **(A). (C–E)**, Quantification of resonant parameters at hyperpolarized and depolarized potentials for identified non-resonant neurons recorded in TTX and TTX/XE991 (*n* = 8). Mann-Whitney paired comparison **(C)**, Two-way repeated measures ANOVA **(D,E)**. ^*^P < 0.05, ^**^P < 0.01, ^***^P < 0.001.

**Figure 4:**
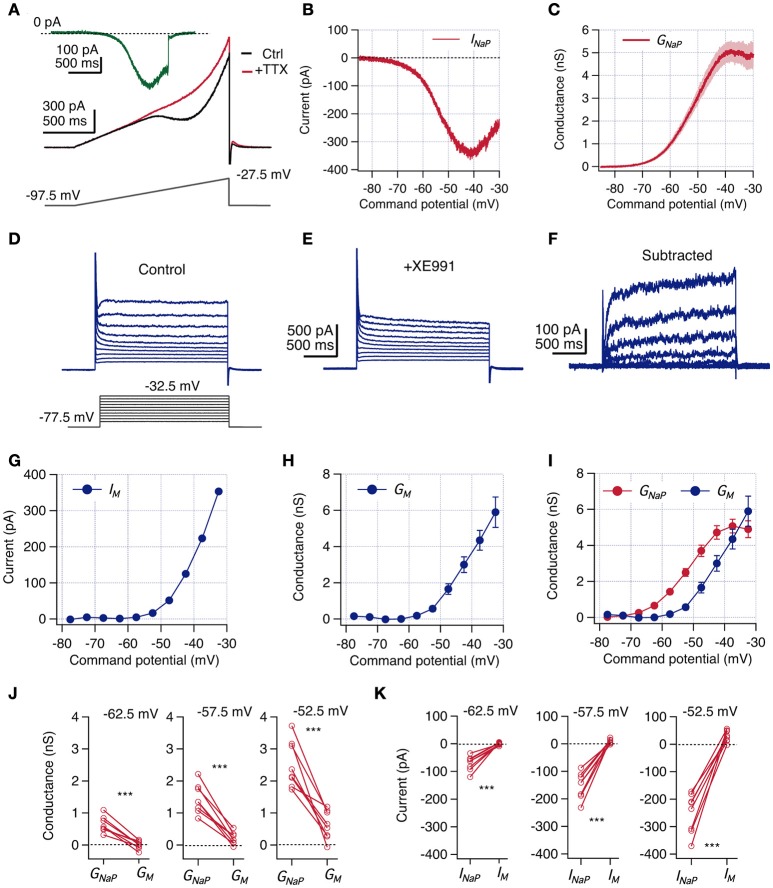
***I***_**NaP**_ **magnitude is larger than ***I***_**M**_ at perithreshold potentials**. Voltage clamp experiments were designed to measure *I*_NaP_ and *I*_M_ consecutively in the same CA1 neurons. **(A)** A voltage ramp protocol was used to isolate *I*_NaP_ by a subtraction method. The slow depolarizing ramp (46.6 mV/s) inactivates transient Na^+^ current in control condition (black) but allows the induction of the persistent current. After addition of 1 μM TTX *I*_NaP_ is blocked (red). Isolated *I*_NaP_ trace is obtained by subtraction of these two recordings (green trace). **(B)** Representative whole-cell *I*_NaP_ vs. command potential curve. **(C)** Average *G*_NaP_ vs. command potential curve calculated from whole-cell currents curves divided by the driving force (*V*_*m*_-*E*_*Na*_, *n* = 13). **(D,E)** Protocol used to measure *I*_M_. Neurons were held at −87.5 mV and a family of 2 s depolarizing squared voltage pulses were used to explore potentials between −77.5 and −32.5 with 5 mV steps, at control conditions **(D)** and after bath addition of 10 μM of XE991 **(E)**. **(F)** *I*_M_ traces are isolated by subtracting recordings in XE991 from those in control condition. **(G)** Representative whole-cell *I*_M_ vs. command potential obtained from **(F)** (current was measured as the average of the last 50 ms of the pulse response). **(H)** Average *G*_M_ vs. command potential curve (*n* = 13). Individual conductance curves were obtained dividing *I*_M_ by *V*_*m*_-*E*_*K*_. **(I)** Overlap of *G*_NaP_ and *G*_M_ curves shown in C and H, respectively. Continue *G*_NaP_ curve was quantized extracting values at same potentials explored for *G*_M_. **(J)** Paired comparison of *G*_NaP_ and *G*_M_ obtained at three near threshold potentials (*n* = 8). **(K)** Paired comparison of *I*_NaP_ and *I*_M_ calculated using reversal potentials from current clamp condition (*n* = 8). Two-way repeated measures ANOVA, ^***^P < 0.001.

Statistical analysis was performed in GraphPad Prism 6.07 (GraphPad Software, Inc, USA). Group data is presented as the mean ± standard error together with the sample size of cells (n). For data with normal distribution (as *Z*_max_, resting potential, *R*_in_, *G*_max_, *G*_(V)_, or *I*_(V)_) we used different parametric tests. When data structure was a single variable measured at different membrane potentials and we needed to compare non-resonant vs. resonant cells (Figure 1) or non-resonant cells in control conditions vs. drug (**Figures 3**, **4**), or the firing probability as function of stimulation frequency in the different experimental conditions (**Figures 6**, **7**), we used two-way repeated measures ANOVA with Holm-Šídák's multiple comparison test. When comparing data at a single membrane potential between non-resonant and resonant cells we used Student's *t*-test (Figure 2). When comparing data at different conditions and a single membrane potential (**Figures 6**, **7**) we used one-way ANOVA with matched samples and Tukey's multiple comparison tests.

When comparing Q or Q′ values we used a non-parametric Mann-Whitney rank test for unpaired data (Figures 1, 2), Wilcoxon rank test for paired data (Figure 3) and Friedman test with Dunn's multiple comparison test to evaluate the effect of PHT and dynamic clamp manipulations (**Figures 6**, **7**). Most statistical tests were two-tailed with exception of those comparing non-resonant vs. resonant data in Figure 2. We used α = 0.05 as critic value.

### Drugs

Drugs were bath-applied at the following final concentrations: 10 μM 6-cyano-7-nitoquinoxaline-2,3-dione (CNQX; AMPA-type glutamate receptor antagonist), 100 μM d-2-amino-5- phosphonovaleric acid (APV; NMDA-type glutamate receptor antagonist), 100 μM picrotoxin (PTX; GABA_*A*_ receptor blocker), 1 μM tetrodotoxin (TTX; voltage-dependentNa^+^ channel blocker), 10 μM XE991 (KCNQ channel blocker), 60 μM phenytoin (PHT, *I*_NaP_ blocker). Drugs were obtained from Sigma, except for XE991 purchased from Tocris, and TTX that was obtained from Alomone Labs.

## Results

### A small fraction of CA1 pyramidal neurons display perithreshold resonance in slices

Our first approach for investigating the perithreshold behavior of pyramidal neurons was to measure their voltage response to ZAP stimulation under whole cell current clamp. We set the ZAP amplitude to produce a peak-to-peak voltage oscillation of ~5–8 mV, while neurons were maintained at different subthreshold potentials by the injection of a stable holding current; for simplicity, we will refer to this potential as “holding potential.” As expected, all CA1 pyramidal neurons displayed resonance at hyperpolarized potentials due to the presence of *I*_*h*_ (Hu et al., 2002; see below). However, these same neurons presented different behaviors at the perithreshold region, just below the spike threshold. We found that 21 out of 26 of recorded neurons (~80%) behaved as a pure low -pass filters, reaching an average perithreshold holding potential of −63.6 ± 2.8 mV. In contrast, the remaining ~20% of them showed a more depolarized perithreshold potential of −62.3 ± 0.6 mV (*P* = 0.014) and expressed a strong frequency preference at theta range (Figure 1A). After impedance analysis, we found that the larger group of neurons presented a *Q* = 1 and *f*_*R*_ of 0.5 Hz, characteristic of non-resonant neurons (Koch, 1984; Hutcheon et al., 1996). The other group displayed a strong resonant profile, with a Q of 1.24 ± 0.09 and a *f*_*R*_ at 3.5 ± 1.6 Hz (Figures 1A–C and raw data in Figure S1), proper of a *I*_M_-dependent resonance (Hu et al., 2002). Those neurons presenting non-resonant behavior also displayed a higher *Z*_max_, in agreement with a lack of activated *I*_M_ (Figures 1A–C, raw data in Figure S1). Thus, here we will refer to CA1 neurons as resonant or non-resonant according to their perithreshold behavior.

As mentioned, voltage responses at −85 mV after ZAP stimulus were similar in the two groups of cells, displaying strong *I*_h_-dependent resonance without differences in Q value, *f*_R_, or *Z*_max_ (Figures 1A–C, raw data in Figure S1). When stimulus was applied at −70 mV both groups of neurons showed reduced resonant behavior, together with similar resonant parameters (Q, *f*_R_, and *Z*_max_, Figures 1A–C, raw data in Figure S1).

To further study the transition from subthreshold to suprathreshold potential, we applied ZAP stimuli just overcoming action potential threshold and quantified the firing probability as a function of frequency (see Section Methods). We found that non-resonant neurons regularly started to fire during the first depolarizing incursion, occurring at 1.2 Hz (Figure 2A upper panel). Resonant neurons displayed higher firing probability at theta range (4–6 Hz), accompanied by a clear attenuation of firing at lower frequencies (Figure 2B). To compare impedance profiles at this suprathreshold region we calculated the Q′ value, using as a reference the impedance at 1 Hz (see Section Methods). While non-resonant neurons maintained a Q′ value near 1 (1.06 ± 0.02) with an average *f*_*R*_ at 1.7 ± 0.2 Hz, resonant neurons displayed a stronger Q′ value (1.35 ± 0.06) with an average *f*_*R*_ at 4.5 ± 0.2 Hz (Figures 2C,D). The peak impedance was higher for non-resonant (231.3 ± 22.4 MΩ) than in resonant (144 ± 22.4 MΩ) neurons, while the holding potential necessary to reach spike threshold with ZAP protocol was more hyperpolarized in non-resonant (−62.48 ± 0.7 mV) than in resonant (−60.0 ± 0.8 mV) neurons (Figures 2E,F).

Since a more depolarized action potential threshold would depolarize the perithreshold region, we measured the spike threshold using a depolarizing current ramp (see Section Methods). We obtained a slightly more depolarized value for resonant neurons, but without reaching statistical significance (−53.6 ± 0.7 mV vs. −52.2 ± 1.1 mV, *P* = 0.19).

### The expression of perithreshold M-resonance is occluded in most neurons by *I*_NaP_ –driven low frequency firing

The described difference in perithreshold behavior of pyramidal neurons suggests that the conductances involved in resonance in this voltage range, *I*_NaP_ and *I*_M_, may have different levels of activation in the two populations.

To evaluate this possibility, we explored the ZAP-induced voltage response at depolarized potentials (up to −45 mV with 5 mV steps) in non-resonant neurons in presence of the selective Na^+^ channel blocker tetrodotoxin (TTX) to avoid spike generation. Interestingly, at voltages above −55 mV all studied neurons displayed strong resonant behavior, with Q values higher than 1.4 and *f*_*R*_ above 4 Hz (Figure 3), even though they were non-resonant near −60 mV. To corroborate that the observed resonant behavior was consequence of *I*_M_ activation we bath-applied the selective M-channel blocker XE991, which consistently eliminated resonance at depolarized potentials, but not at hyperpolarized potentials (Figure 3).

These experiments show that M-resonance consistently appears in all neurons at voltages more depolarized than −55 mV (see also Hu et al., 2002), which in most cases is above the spike threshold.

The difference in the holding voltage level at which resonant and non-resonant cells start firing (in a frequency-selective or unselective way, respectively; see Figure 2F) might reflect a different voltage window for interaction between *I*_NaP_ and *I*_M_, with the consequent generation of resonant and non-resonant behaviors. To test this possibility, we investigated the degree of interaction between both currents in the same neuron.

### Measurement of somatic *I*_NaP_ and *I*_M_

We measured *I*_NaP_ under voltage clamp by eliciting whole cell currents with a slow voltage ramp (30 mV/s) (Magistretti and Alonso, 1999) to allow *I*_*NaT*_ inactivation, both in control condition and after the application of 1 μM TTX (Figure 4A). Then, *I*_NaP_ was obtained by digitally subtracting the recording in TTX from the control condition (Figure 4B). By this procedure, we obtained a persistent TTX-sensitive current that displayed non-linear voltage dependence, activated at command potentials above −70 mV and reached peak amplitude near −40 mV (Figure 4B). To achieve a better voltage clamp in the presence of Na^+^ currents, in these experiments we decreased the driving force by reducing extracellular Na^+^ concentration (see Section Methods). Therefore, to compare voltage-clamp results with the observations made in current clamp conditions we calculated the persistent Na^+^ conductance (*G*_NaP_) by dividing the measured current by its driving force (*V*_m_–*E*_Na_). The voltage-dependent activation of *G*_NaP_ was characterized by fitting a sigmoidal curve to each trace (see Section Methods), obtaining a voltage for half activation (*V*_0.5_) of −52.2 ± 1.0 mV, a slope constant of 4.9 ± 0.4 mV and a maximal conductance plateau of 5.3 ± 0.5 nS reached at voltages above −40 mV (*n* = 13; Figure 4C). These values are in agreement with previous investigations performed in dissociated CA1 neurons (French et al., 1990) and acute slices (Vervaeke et al., 2006), thus supporting the quality of our measurements. These results show that at perithreshold potential (near −58 mV) a substantial amount of *G*_NaP_ is active, thus strongly driving cells toward spike threshold.

Since *I*_M_ is absent at hyperpolarized potentials and activates with depolarization (Halliwell and Adams, 1982), we evoked whole-cell currents by 2 s depolarizing steps from a holding potential of −87.5 mV to voltages between −77.5 and −32.5 mV, with increments of 5 mV. To measure *I*_M_ in the same cells in which we previously measured *I*_NaP_, we applied this voltage protocol in the presence of TTX as the control condition. The described voltage protocol elicited a sustained whole-cell outward current that contained a combination of several ionic currents (Figure 4D). To isolate *I*_M_ we bath-applied 10 μM of the selective blocker XE991 and repeated the protocol after 5–8 mins (Figure 4E), obtaining the XE991-sensitive current by digital subtraction (Figure 4F).

The isolated current showed a slow activation constant (60–200 ms) with no inactivation (Figure 4F), in agreement with the described *I*_M_ kinetics (Shah et al., 2002). The I-V curve shows that the isolated current is inactive at resting potential and begins to activate above −55 mV, increasing monotonically with voltage and reaching ~ 400 pA at −32.5 mV, the maximal voltage tested (Figure 4G). We computed the *G-V* curve dividing the I-V trace by the driving force for K^+^, obtaining a *G*_M_ curve that appears above -60 mV and reaches ~ 6 nS at −32.5 mV (Figure 4H).

### Comparing perithreshold behavior of *I*_NaP_ and *I*_M_

Once we measured the voltage dependence of *I*_NaP_ and *I*_M_ under the same conditions and in the same cells, we were able to estimate their relative activation levels near perithreshold potential. Interestingly, the comparison of both curves shows that at all voltages between −62.5 and −42.5 mV, *G*_NaP_ is larger than *G*_M_ (Figure 4I). A closer inspection to activated conductances at perithreshold potentials (−62.5, −57.5, and −52.5 mV) shows that in all paired recordings the amount of *G*_NaP_ always exceeds *G*_M_; with *G*_NaP_ ranging from 0.6 to 2.5 nS, whereas *G*_M_ only reaches a modest average activation of 0.6 nS at −52.5 mV (See paired values in Figure 4J).

Since at perithreshold potentials the driving force for Na^+^ is higher than for K^+^, is expected that the magnitude of the difference between *I*_NaP_ and *I*_M_ will be even higher than the difference of their respective conductances. With the driving force calculated from the ionic concentrations in our current clamp experiments (see Section Methods) we obtained that *I*_NaP_ is around −150 pA at spike threshold levels, while *I*_M_ is only 9 pA, maintaining this paired relative difference for each recorded neuron (See paired values in Figure 4K). These quantifications confirm the reduced contribution of *I*_M_ in the perithreshold region of non-resonant neurons.

### Exploring perithreshold behavior with computer simulations

To evaluate the expected perithreshold behavior of neurons recorded under voltage clamp we constructed a conductance-based computational model to be applied to each neuron according to their own measured *I*_NaP_, *I*_M_, and capacitance (see Section Methods). To gain detail and precision in the computer simulations we fed the model with the specific intrinsic parameters of each cell, instead of using the average values from a diverse population. As we were interested in evaluating perithreshold frequency preference we did not include in the model the conductances related to action potential generation.

**Figure 5:**
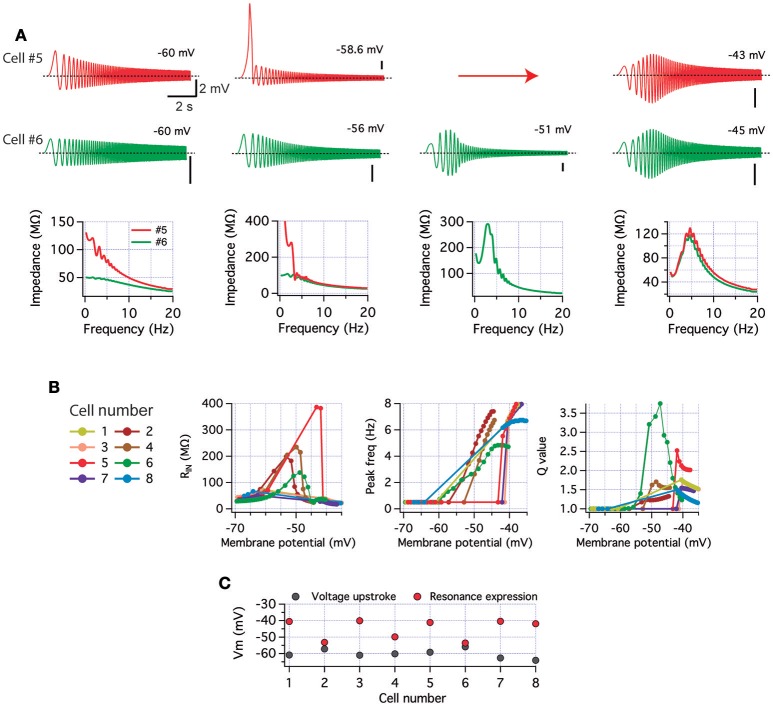
**Experimentally measured levels of ***I***_**NaP**_ and ***I***_**M**_ reproduce perithreshold variability in a model of CA1 pyramidal neuron**. Computer simulations of cell excitability were performed using a conductance-based model to explore perithreshold dynamics with the values for *I*_NaP_ and *I*_M_ measured in voltage-clamp conditions (Figure 4). The model did not include Hodgkin and Huxley conductances (see Section Methods), thus spikes are absent. **(A)** Voltage response (top) and impedance profile (bottom) of two cells at different depolarized potentials (the arrow indicates a discontinuity in the accessible voltages, see main text). Cells present different non-resonant or resonant properties depending on potential (see text). Calibration bars are 2 mV and 2 s. **(B)** Summary of input resistance, peak frequency, and Q value from all simulated neurons as a function of holding membrane potential (from −70 to −35 mV). **(C)** Membrane potential values for *I*_NaP_-driven upstroke (gray) and for the emergence of resonance (red) in each cell.

We explored the voltage response to ZAP stimuli for membrane potentials maintained between −70 and −35 mV through injection of a holding current, thus simulating typical current-clamp experiments. Recreated neurons reproduced the variable behavior observed in current clamp experiments, as represented by cells #5 and #6 in Figure 5. Cell #5 displays non-resonant behavior between −70 and −60 mV. However, around the presumed perithreshold potential (near −58 mV), a voltage upstroke of about 10 mV is observed at the first and slowest oscillation of the ZAP, indicating a significant activation of *I*_NaP_ (note that a real spike is not seen because Hodgkin & Huxley conductances were omitted in the model; Figure 5A, red). Interestingly, at this point cell #5 holding potential became unstable, i.e., small increments in holding current generated a step-like transition from −58.6 to −43 mV, a potential at which the neuron stabilizes due to the compensatory effect of *I*_M_ activation, which also generates a strong resonant behavior.

In contrast, cell #6 displays a slight resonant behavior even at presumed perithreshold potentials. Further, current injection produces a gradual depolarization of holding potential accompanied by an increase in resonant behavior, instead of the discontinuous change observed in cell #5 (Figure 5A green). In this second example, the small amount of *I*_M_ activated at perithreshold potential allows the expression of resonance by decreasing voltage oscillations at lower frequencies and preventing the strong depolarization driven by *I*_NaP_ in the previous case. Further, co-activation of *I*_NaP_ and *I*_M_ by depolarization allows a balanced interplay between both currents that prevents voltage instabilities as in cell #5, producing a strong resonance with depolarization. The impedance profiles for both neurons also show the different behavior at each holding potential (Figure 5A bottom). Note that while responses are different at perithreshold potential, after depolarization to near −40 mV their impedance profiles are similar. Figure 5B shows the input resistance, peak frequency and resonance strength values against membrane potential for all recreated neurons. An important quality control of the simulations is the reproduction of the increase in input resistance caused by the amplifying effect of *I*_NaP_, and the rise of Q value attained by *I*_M_ activation. While the general trend was to switch from non-resonant to resonant behavior upon increasing depolarization, resonance appears at different membrane potentials and in most cases following a membrane potential discontinuity as described above (note that this behavior is observed even when holding current increments are as small as 5 pA). As indicated by peak frequency and Q plots in Figure 5B, transition from non-resonant to resonant behavior is a heterogeneous process with a different voltage point for each neuron. Some neurons transit to depolarized potentials from values as hyperpolarized as −60 mV (cells #1, 3, 7, and 8, Figure 5B). Certainly, this transition is visible in our model due to the absence of spiking activity. In physiological conditions the transition to resonant behavior in most cases is occluded by the activation of the spiking machinery (as seen in Figures 1, 2). It should be noted that the experiments with TTX as those in Figure 3 are also not comparable with these simulations as this drug not only abolishes action potentials but also block *I*_NaP_.

To predict the perithreshold behavior of recreated cells, we compared the membrane potential at which *I*_NaP_ activation drives the depolarizing voltage discontinuity (when present) with the membrane potential where resonance is expressed (Q > 1). If these two values are close to each other and more hyperpolarized than our measured spike threshold (near −52 mV, Figure 2) we considered that neuron as putative resonant (Figure 5C). According to this criterion, cells #2 and #6 are putative resonant neurons, while the other 6 neurons are presumed to be non-resonant, in agreement with the abundance of both perithreshold behaviors previously presented (Figure 1).

The computational exploration of voltage responses using experimentally measured combinations of *I*_NaP_ and *I*_M_ that constitutes a sample group in the physiological variability of these conductances, supports the idea that the expression of perithreshold resonance depends on their relative magnitudes.

### Inducing resonant behavior by blockade of *I*_NaP_

**Figure 6:**
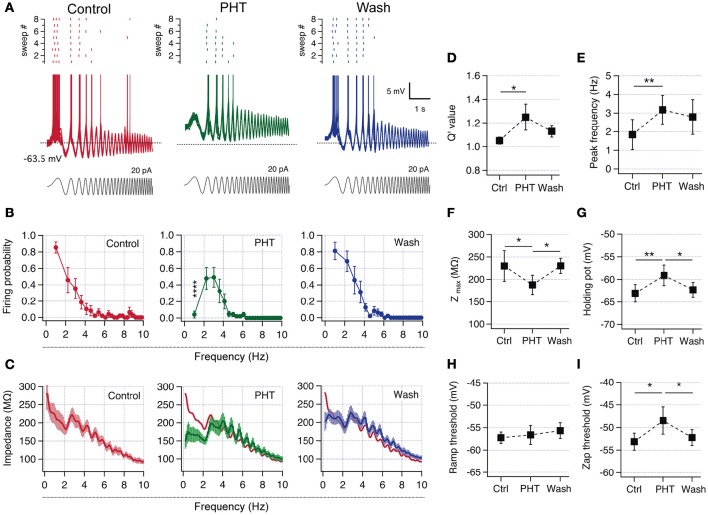
**Pharmacologic reduction of ***I***_**NaP**_ switched CA1 pyramidal neurons from non-resonant to resonant. (A)** Superposition of 8 consecutive suprathreshold voltage responses in a non-resonant CA1 pyramidal neuron under ZAP stimulation (gray), in consecutive control (red), 60 μM phenytoin (PHT, green), and wash-out (blue) conditions (spikes were cut at −43 mV). The raster plot of the spiking activity at each condition is shown at the top of each family traces. **(B)** Average firing probability curves of recordings from 6 initially non-resonant neurons (^*^ indicate difference between PHT and control and wash out conditions). **(C)** Average suprathreshold impedance profiles for the three conditions. The red trace in the PHT graph is the average curve from control condition. **(D–I)** Average parameters for the three conditions. Q′ value **(D)**, peak frequency **(E)**, peak impedance **(F)**, holding potential at which transition to spiking occurs **(G)**, spike threshold measured with a depolarizing current ramp **(H)** and spike threshold of spikes fired during ZAP stimulation **(I)**. Two-way ANOVA **(B)**, Friedman test **(D)**, one-way ANOVA **(E–I)**. ^*^P < 0.05, ^**^P < 0.01, ^****^P < 0.0001.

Since we confirmed that all recorded pyramidal neurons have the intrinsic mechanism to display resonant behavior, we now explored if a partial block of *I*_NaP_ can produce resonance in non-resonant neurons. Therefore, we characterized perithreshold behavior and firing preference in control condition, after the addition of 60 μM of the *I*_NaP_ blocker and antiepileptic drug phenytoin (PHT; Chao and Alzheimer, 1995), and after washing out the drug. In control conditions neurons were non-resonant, with a high voltage response and firing probability at the slowest oscillations (Figure 6A). When the *I*_NaP_ blocker was added, was necessary to inject more DC current in order to depolarize neurons up to perithreshold potential, compensating the blocking of a depolarizing current (not shown). Moreover, the reduction of *I*_NaP_ also shifts to depolarized values the holding potential necessary to trigger spikes, consistent with a reduction in the intrinsic depolarizing force (note the more depolarized holding potential in Figures 6A,G). These changes are accompanied by modifications in the spiking behavior, displaying an attenuated response at low frequencies and a maximal firing probability in the theta range, thus becoming a resonant neuron (Figure 6A middle/green). When the drug was washed out the neuron recovered its original non-resonant dynamics (Figure 6A, *n* = 6). Figure 6B shows the average firing probability curves, indicating that cells moved from a robust low-pass filter behavior at control condition to a band-pass filter pattern when a fraction of *I*_NaP_ is blocked. The effect is almost completely reversed upon blocker removal. The suprathreshold impedance profile in control condition has a low-pass filter shape with a peak impedance of 233.5 ± 31 MΩ at 1 Hz. Note however that the curve displays a hump near 4 Hz that breaks the monotonic drop of impedance as frequency increases (Figure 6C, left). In presence of phenytoin the impedance at 1 Hz falls below 200 MΩ evidencing the band-pass filter pattern, with the impedance peak at the same frequency of the hump observed in control conditions (see overlapped curves in Figure 6C, middle). This change is partially reversed after washing out the drug, and the impedance at 1 Hz grew above 200 MΩ. Regarding resonant parameters, after partial block of *I*_NaP_ Q′ rises from 1.05 ± 0.03 to 1.7 ± 0.7, while *f*_*R*_ increases from 1.27 ± 0.10 Hz to 3.6 ± 0.5 Hz, consistent with a resonant behavior generated by *I*_M_ (Figures 6D,E). In agreement with the amplifying effect of *I*_NaP_, phenytoin addition produced a 20% drop of peak impedance (Figure 6F).

The partial block of *I*_NaP_ produced a raise in the potential at which transition to spiking occurs from −63.1 ± 1.5 to −59 ± 1.8 mV (Figure 6G), while the spike threshold measured with a voltage ramp was not modified. However, the spike threshold measured when spikes were fired during suprathreshold oscillations suffered a depolarizing shift from −54.1 ± 1.4 to −49.5 ± 2.3 mV (Figures 6H,I). The transformation of perithreshold behavior induced by phenytoin was partially reverted after drug washout.

These results confirm that non-resonant hippocampal neurons are able to change their firing frequency preference to a resonant behavior by a moderate reduction in *I*_NaP_.

### Setting frequency selectivity through a sliding balance between *I*_NaP_ and *I*_M_

Our following goal was to evaluate the effect of changing the perithreshold balance of *I*_NaP_ and *I*_M_ in non-resonant neurons using dynamic clamp (Dorval et al., 2001). We produced a virtual knock-down of *I*_NaP_ by injecting a negative *I*_NaP_ (−*I*_NaP_) to neutralize a fraction of total endogenous *I*_NaP_ (see Section Methods). This strategy is comparable to the previous pharmacological blockade of *I*_NaP_ but now with the possibility to determine the conductance change necessary to produce the switch in perithreshold behavior.

To investigate whether non-resonant neurons are able to resonate with their natural intrinsic levels of *I*_NaP_ in specific conditions, we used dynamic clamp to incorporate a virtual *I*_M_ (+*I*_M_) based on the activation curve previously obtained by voltage-clamp experiments, but shifting the voltage sensitivity toward hyperpolarized potentials to increase the voltage window of interaction with *I*_NaP_ (see Section Methods).

**Figure 7:**
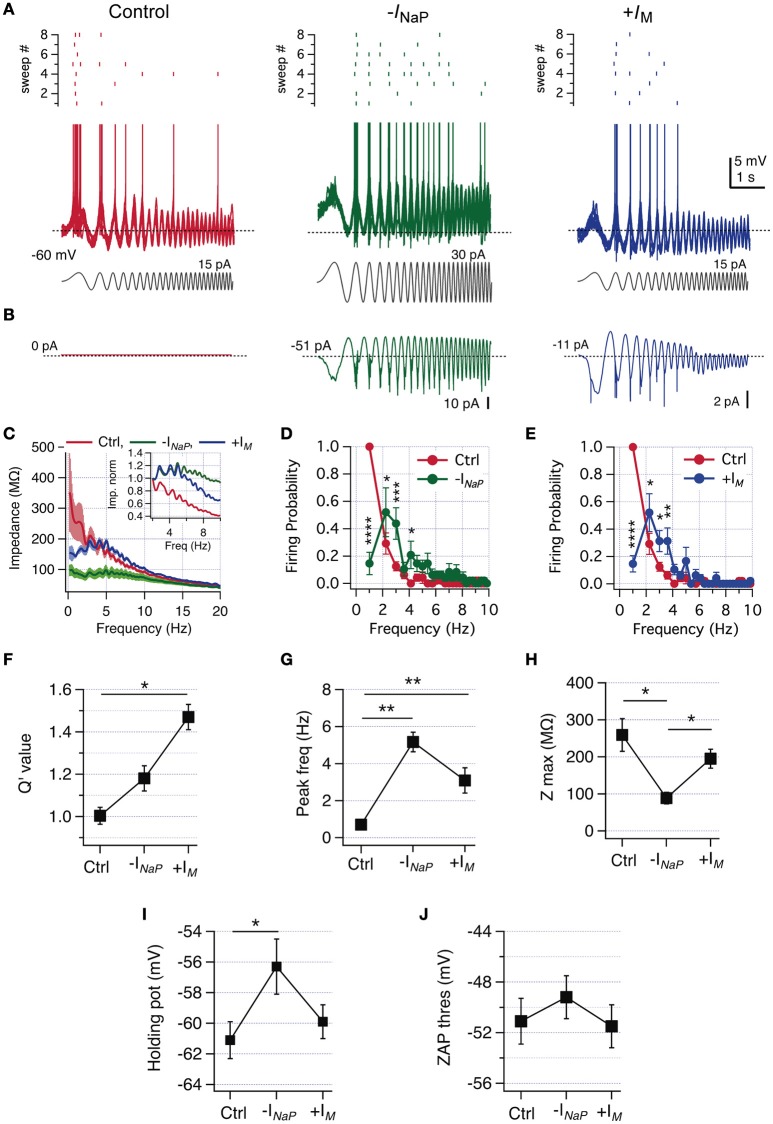
**Reduction of ***I***_**NaP**_ or increase of ***I***_**M**_ by dynamic-clamp produces a switch from non-resonant to resonant behavior in CA1 pyramidal neurons. (A)** Superposition of 8 consecutive suprathreshold voltage responses of a non-resonant CA1 pyramidal neuron under ZAP stimulation (gray), in control condition (red) and after virtual canceling of *I*_NaP_ (-*I*_NaP_, green) or *I*_M_ increase (+*I*_M_, blue), by means of dynamic clamping (spikes were cut at −43 mV). The raster plot of the spiking activity at each condition is shown at the top of voltage traces. **(B)** Average dynamic current injected at each condition, dotted line indicate steady current level. **(C)** Average impedance profile in control (red), –*I*_NaP_ (green trace) and +*I*_M_ (blue trace) conditions. Inset, zoom of average curves normalized to the impedance value at 2 Hz (see Section Methods). **(D)** Average firing probability curves for control (red) and –*I*_NaP_ (green) conditions. **(E)** Average firing probability curves for control (red) and +*I*_M_ (blue) conditions (*n* = 6). **(F–J)** Q′ value **(F)**, peak frequency **(G)**, peak impedance **(H)**, holding potential for transition to spikes **(I)** and the spike threshold of the spike fired under ZAP stimulation **(J)**. Two-way repeated measures ANOVA **(D,E)**, Friedman test with Dunn's multiple comparison **(G)**, one-way ANOVA **(H–J)**. ^*^P < 0.05, ^**^P < 0.01, ^***^P < 0.001, ^****^P < 0.0001.

We first characterized the frequency preference of a neuron in control condition, corroborating the lack of frequency preference in voltage response and firing probability (Figure 7A, left). Then we injected −*I*_NaP_, beginning with a maximal conductance (*G*_NaPMax_) of 2 nS. This amount was enough to observe resonance in three out of six neurons (Figures 7A–C). In the other three neurons, it was needed to increase the amount of −*I*_NaP_ to 4.5 nS in one case and to 9 nS in the other two cells to obtain a similar response. Despite the differences in the amount of injected −*I*_NaP_ (canceling an average of −147.6 ± 61.9 pA of endogenous *I*_NaP_, not shown), it was possible to induce an equivalent resonant behavior in all 6 neurons, as expressed in the impedance profile (Figure 7C) and in the drop of firing probability at low frequencies (Figure 7D). Reduction of *I*_NaP_ modified Q′ from 1.0 to 1.18 ± 0.06, but without reaching statistical significance (*P* = 0.12, Figure 7F) and shifted *f*_*R*_ from 0.7 to 5.2 Hz (Figure 7G). In agreement with the amplifier role of *I*_NaP_, its neutralization reduces peak impedance from 259 ± 44.1 to 88.8 ± 15.6 MΩ (Figures 7C,H).

The injection of −*I*_NaP_ depolarizes the holding potential just below threshold from −61.1 ± 1.2 to −56.3 ± 1.8 mV (Figure 7I) as in phenytoin experiments. Importantly, the modification of *I*_NaP_ did not alter drastically the action potential threshold measured during oscillatory stimulation (Figure 7J).

After evaluating the effect of −*I*_NaP_ injection we explored the consequences of injecting +*I*_M_ in the same cells. It was needed to shift the *V*_0.5_ of the activation curve from −39 to −49 mV (*n* = 3), −54 mV (*n* = 1) or −59 mV (*n* = 2), to achieve a critical amount of virtual *I*_M_ allowing to change the perithreshold behavior from non-resonant to resonant (average +*I*_M_ current injected during voltage oscillations was −20.1 ± 7.3 pA, not shown). As expected, intrinsic *I*_NaP_ interacted with +*I*_M_ to induce a perithreshold resonant behavior able to translate frequency preference to spiking regime, with a reduction in firing probability at 1.2 Hz from 1.0 to 0.15 ± 0.07 (Figure 7E). +*I*_M_ injection only reduced impedance at frequencies below 4 Hz (Figure 7C) and presented a trend to reduce peak impedance (Figure 7I). The combination of endogenous high levels of *I*_NaP_ plus a critical amount of +*I*_M_ produces a strong resonant behavior, rising Q′ and shifting average *f*_*R*_ from 0.7 ± 0.2 to 3.1 ± 0.4 Hz (Figures 7F,G). As expected from the unaltered *I*_NaP_, in the +*I*_M_ condition resonant behavior develops without altering the perithreshold holding membrane potential, nor the spike threshold (Figures 7I,J).

Taken together, these results show that the behavior of CA1 pyramidal neurons can be transformed from non-resonant to resonant by changing the relative contributions of *I*_NaP_ and *I*_M_.

## Discussion

Here we report that CA1 pyramidal neurons present two different types of behaviors when stimulated with an oscillatory current of variable frequency that spans the theta range (0–20 Hz). A small population (20%) of them express perithreshold resonance and fire selectively at theta frequency (4–6 Hz), while the remaining ones (80%) behave as low-pass integrators, i.e., without theta frequency tuning (Figure 1). It is important to note that these two behaviors are expressed independently of the hyperpolarized *I*_h_-resonance (Hu et al., 2002), which was always present (Figure 1). These non-resonant neurons preserve their behavior when depolarized until reaching spike threshold, firing spikes with higher probability at the lowest frequencies of stimulation range (Figure 2). However, all of these non-resonant cells can display *I*_M_-driven resonance when depolarized in presence of the Na^+^ channel blocker tetrodotoxin (TTX), suggesting that in regular conditions spike firing at the lowest frequencies precludes the expression of resonance (Figure 3). In fact, paired measurement of *I*_NaP_ and *I*_M_ in the same cells showed that at all subthreshold potentials the activation level of *I*_M_ is very low, while *I*_NaP_ is activated enough to depolarize neurons toward spike threshold (Figure 4). Computer simulations using the parameters measured in each specific cell in our voltage clamp experiments support the idea that CA1 pyramidal neurons display heterogeneous contributions of *I*_NaP_ and *I*_M_ and that the expression of perithreshold resonance depends on the interplay between these two conductances (Figure 5). Consistent with this possibility, a partial block of *I*_NaP_ with phenytoin or its reduction by dynamic clamp allowed us to change the behavior of non-resonant neurons to resonant (Figures 6, 7), demonstrating that different activation levels of *I*_NaP_ can modulate the perithreshold and spiking behavior of a neuron. On the other hand, displacing the activation range for *I*_M_ toward hyperpolarized potentials using dynamic clamp, also transforms non-resonant neurons into resonant (Figure 7).

The contribution to single cell excitability of *I*_NaP_ and *I*_M_ has been extensively described in central neurons, but mostly separately. *I*_NaP_ contributes to repetitive firing and to generate rhythmic subthreshold membrane potential oscillations, and also plays a general role as an amplifier current increasing after potential hyperpolarization, synaptic voltage responses (EPSP and IPSP) and promoting resonance (Schwindt and Crill, 1995; Stuart and Sakmann, 1995; Parri and Crunelli, 1998; Stuart, 1999; D'Angelo et al., 2001; Hu et al., 2002; Sanhueza and Bacigalupo, 2005; Vervaeke et al., 2006; Tazerart et al., 2008; Boehlen et al., 2013; Yamada-Hanff and Bean, 2013; Vera et al., 2014). *I*_M_ is the main current involved in frequency accommodation, control of cell excitability, and it has also been described as a current involved in resonance (Hu et al., 2002, 2007; Peters et al., 2005; Lawrence et al., 2006; Shah et al., 2008; Leão et al., 2009; Hönigsperger et al., 2015).

Prescott et al. (2008) showed that CA1 pyramidal neurons behave like integrators under *in vitro* conditions (characterized by low synaptic input) and that under *in vivo*-like conditions (under recreated high synaptic stimulation) they switch to resonant behavior by an extrinsically-driven increase of *I*_M_ activation and a secondary depolarization of spike threshold. This switch is a mixed mechanism including intrinsic and synaptic properties and does not explain our observation of resonant behavior under *in vitro* conditions (Hu et al., 2002). Moreover, a recent investigation reported that under muscarine stimulation reducing *I*_M_ and other cationic conductances, *I*_NaP_ drives rhythmic spontaneous firing in CA1 pyramidal neurons (Yamada-Hanff and Bean, 2013), highlighting the relevance of the interaction between *I*_NaP_ and *I*_M_ in controlling cell activity at perithreshold voltage range.

The perithreshold region is an unstable and highly non-linear zone where these currents have opposed effects on membrane potential, with *I*_NaP_ producing depolarization toward spike threshold and promoting an integrator behavior, and *I*_M_ hyperpolarizing the neuron and driving a resonant behavior. It is therefore expected that the precise perithreshold dynamics of CA1 pyramidal neurons should depend on the balance between these two currents. Here we found that in the same conditions and even in the same slice, CA1 pyramidal neurons can behave as non-resonant or resonant relying only on intrinsic properties. We show that at perithreshold potentials *I*_NaP_ displays some degree of variability in activation among the recorded neurons, whereas *I*_M_ is mostly inactive (Figures 4I–K). However, small amounts of activated *I*_M_ will allow resonance expression (Figures 5, 7). For this reason, we propose that the perithreshold behavior of CA1 pyramidal neurons is variable and its specific shape will depend on the relative levels of *I*_NaP_ and *I*_M_ given by their particular values of maximal conductances and voltage sensitivities. Thus, perithreshold behavior of CA1 pyramidal neurons lies on a continuum between two extreme configurations. At one extreme are those neurons in which *I*_NaP_ activation begins at relatively hyperpolarized potentials, setting a steady state perithreshold voltage below activation ranges for *I*_M_ and displaying a strong non-resonant behavior. Whereas in the other extreme are those neurons in which low levels of *I*_NaP_ induce a slow depolarization that inactivates a fraction of *I*_*NaT*_ and also of *I*_NaP_ (Colombo et al., 2013), reaching a more depolarized steady state potential and concomitant shift of spike threshold to positive values (Fernandez and White, 2010). This depolarized perithreshold potential allows *I*_M_ activation and the expression of resonant behavior. According to this, all CA1 pyramidal neurons have the intrinsic ability to display perithreshold frequency tuning, but the expression of this property would depend on whether the level of *I*_NaP_ allows significant *I*_M_ activation before spiking is triggered. Conversely, those neurons where perithreshold levels of *I*_M_ are high enough to counterbalance *I*_NaP_-driven depolarization will filter low frequency oscillations and express resonant behavior.

Our experiments with pharmacology and dynamic clamping demonstrate that reducing the amount of *I*_NaP_ or increasing *I*_M_ is enough to change the behavior of one neuron from non-resonant to resonant. Given the subtle change needed to transform the perithreshold behavior, it is plausible that in the intact brain neurons can switch between these behaviors due to the modulation of current amplitude and/or voltage sensitivity by intracellular second messengers or neuromodulators. In fact, both currents are highly regulated, with *I*_NaP_ being modulated in amplitude and/or voltage sensitivity by PKC (Astman et al., 1998), dopamine receptor activation (Gorelova and Yang, 2000), oxidative metabolism (Hammarstrom and Gage, 1998) and G-protein subunits composition (Ma et al., 1997; Mantegazza et al., 2005), and *I*_M_ being suppressed by activation of muscarine receptors (Delmas and Brown, 2005) and by endocannabinoids (Schweitzer, 2000) or increased by somatostatin (Moore et al., 1988). Moreover, muscarinic receptor activation may directly modulate *I*_NaP_ (Mittmann and Alzheimer, 1998). This degree of modulation may be, at least in part, the reason why some authors have described the behavior of CA1 pyramidal neurons at perithreshold potential as integrators and others as resonators (discussed in Hu et al., 2002). Thus, the observation of distinct excitability profiles within the same cell type is highly expected.

The fact that we found different perithreshold behavior in the same experimental conditions, with the control of intracellular metabolites concentration and the composition of extracellular recording solution, suggests that one possible cause of the differential behavior relies on the specific quantities of Na_V_ and KCNQ channels expressed at each neuron. This is in agreement with the current view of cell excitability, in which the level of expression of each conductance varies from cell to cell, producing heterogeneous mixes of membrane conductances that would shape the specific behavior of a neuron (Destexhe and Marder, 2004; Marder and Goaillard, 2006). Thus, together with the transient modulation of *I*_NaP_ and *I*_M_, there is a structural variability that sets a specific subthreshold behavior of neurons when observed under controlled conditions.

Finally, we propose that CA1 neurons are endowed with the intrinsic ability to switch between non-resonant and resonant behavior through a sliding balance of *I*_NaP_ and *I*_M_. Considering that the behavior of the neurons at perithreshold potentials has a tremendous influence in the way neurons translate subthreshold activity to action potentials, this intrinsic switching mechanism might dynamically tune responsiveness of neurons favoring the processing of specific rhythms. The fact that both currents are highly modulated by intracellular signals and neuromodulators (Moore et al., 1988; Ma et al., 1997; Astman et al., 1998; Hammarstrom and Gage, 1998; Gorelova and Yang, 2000; Schweitzer, 2000; Delmas and Brown, 2005; Mantegazza et al., 2005) can introduce a fast mechanism of perithreshold frequency tuning in CA1 hippocampus favoring selective firing at theta rhythm. Furthermore, since *I*_NaP_ (Stafstrom, 2007) and *I*_M_ (Halliwell, 1986) are expressed in most cortical neurons, the mechanism described here is possibly a more general strategy to control selective firing under oscillatory synaptic input.

## Author contributions

JV and MS conceived and designed the experiments, JV performed the experiments and developed the computational model. JV and JA analyzed the data. JA and MS contributed reagents/materials/analysis tools. JV, JA, and MS wrote the paper.

## Funding

Fondo Nacional de Desarrollo Científico y Tecnológico (FONDECYT) 1140700 (MS), 3150668 (JV) and 1130177 (JA).

### Conflict of interest statement

The authors declare that the research was conducted in the absence of any commercial or financial relationships that could be construed as a potential conflict of interest.

## Supplementary material

Figure S1 **Quantification of Q-value, frequency of preference and peak impedance in CA1 neurons**. Raw data from analysis shown in Figure 1. **(A–C)** Average values (filled circles) vs. membrane potential shown in Figure 1C, with the addition of the data from single experiments (rectangles). Resonant (Res, *n* = 5) and non-resonant (Non, *n* = 21) neurons are defined according to their Q value at depolarized subthreshold potential (Methods). **(D–F)**. In order to facilitate visual analysis of the raw data, we plotted same data as in **(A–C)** defining three ranges of membrane potential: hyperpolarized (Hyp, ~ −85 mV), near resting (Res, ~ −70 mV), and depolarized (Dep, ~ −63 mV), for resonant (Res, *n* = 5) and non-resonant (Non, *n* = 21) neurons.
