# Supplementary figures and images for: Competition between Persistent Na+ and Muscarine-Sensitive K+ Currents Shapes Perithreshold Resonance and Spike Tuning in CA1 Pyramidal Neurons
Source: Front Cell Neurosci. 2017 Mar 8;11:61. doi: 10.3389/fncel.2017.00061 (PMC5340745; doi:10.3389/fncel.2017.00061)

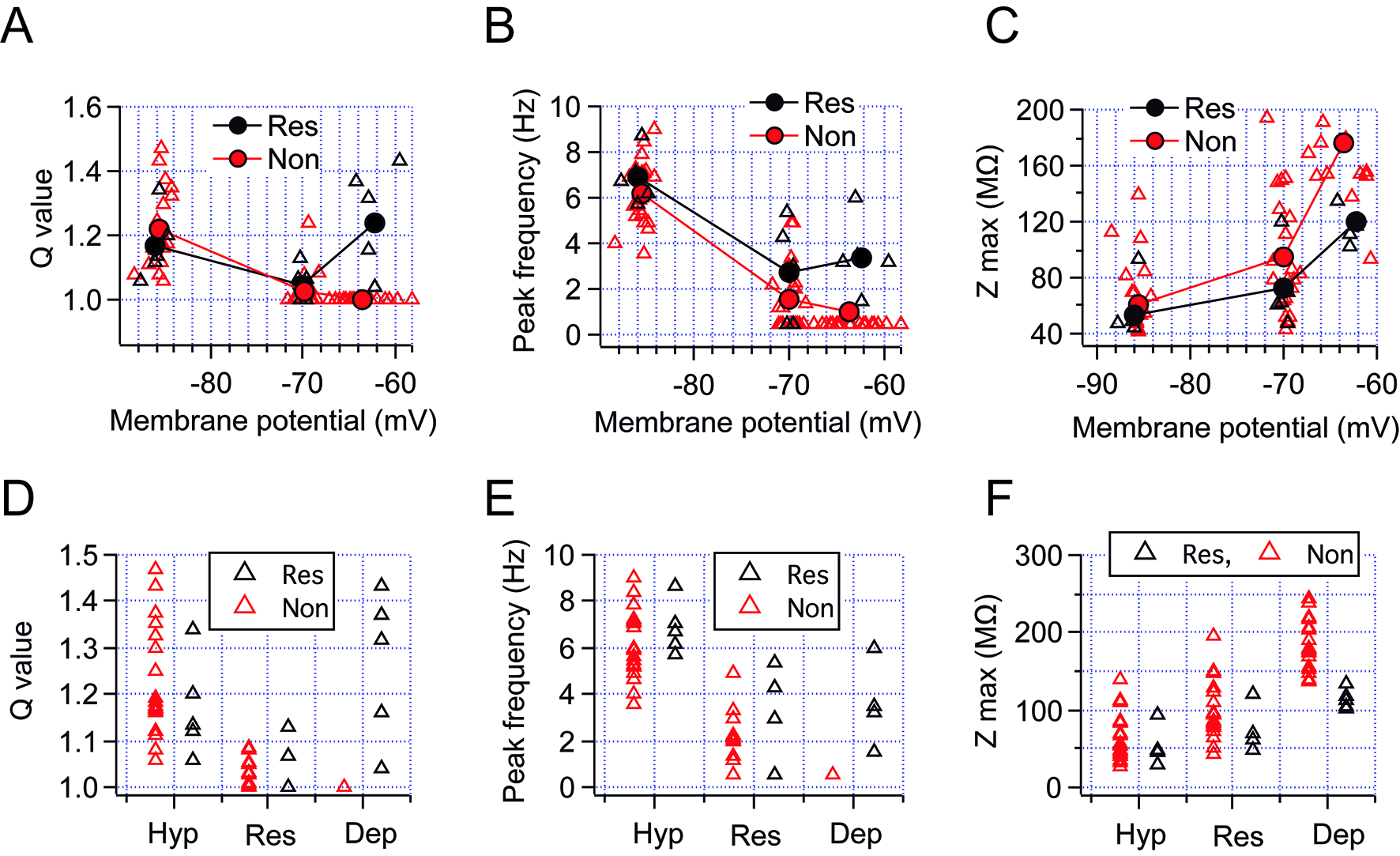

Supplement: Figure S1 — Quantification of Q-value, frequency of preference and peak impedance in CA1 neurons. Raw data from analysis shown in Figure 1. (A–C) Average values (filled circles) vs. membrane potential shown in Figure 1C, with the addition of the data from single experiments (rectangles). Resonant (Res, n = 5) and non-resonant (Non, n = 21) neurons are defined according to their Q value at depolarized subthreshold potential (Methods). (D–F). In order to facilitate visual analysis of the raw data, we plotted same data as in (A–C) defining three ranges of membrane potential: hyperpolarized (Hyp, ~ −85 mV), near resting (Res, ~ −70 mV), and depolarized (Dep, ~ −63 mV), for resonant (Res, n = 5) and non-resonant (Non, n = 21) neurons. [file Image1.TIF]
